# Purslane (*Portulaca oleracea* L.) Extract Alleviates Atherosclerosis in ApoE^−/−^ Mice

**DOI:** 10.1002/mnfr.70449

**Published:** 2026-03-27

**Authors:** Yuehan Wang, Junming Chen, Hua Yu, Yuanshan Yu, Manqin Fu, Wai San Cheang

**Affiliations:** ^1^ Institute of Chinese Medical Sciences State Key Laboratory of Mechanism and Quality of Chinese Medicine University of Macau Macau SAR China; ^2^ Sericultural & Agri‐Food Research Institute Guangdong Academy of Agricultural Sciences/Key Laboratory of Functional Foods Ministry of Agriculture and Rural Affairs/Guangdong Key Laboratory of Agricultural Products Processing Guangzhou China

**Keywords:** atherosclerosis, endothelial cells, inflammation, purslane, vasorelaxation

## Abstract

*Portulaca oleracea* L. (purslane) is a widely cultivated herb with edible and medicinal value. Modern pharmacological studies have shown that purslane has potent anti‐inflammatory effects. However, its potential role in ameliorating atherosclerosis remains unclear. This study aimed to investigate the efficacy of purslane extract in ameliorating atherosclerosis in apolipoprotein E(ApoE) knock‐out (ApoE^−/−^) mice. ApoE^−/−^ mice fed a Western diet were administered either purslane ethanol extract (400 mg/kg/day) or a vehicle for 4 weeks. Furthermore, isolated aortas from C57BL/6J mice were treated with lysophosphatidylcholine (LPC) with or without purslane extract for ex vivo experiments. Oil red O staining, functional study by wire myograph, fluorescence imaging, and Western blotting were performed. Purslane extract normalized plasma lipid profiles, reducing total cholesterol, triglycerides, and LDL while increasing HDL in ApoE^−/−^ mice. It also reduced plasma interleulin‐6 concentration. Purslane extract reduced atherosclerotic plaque area and ROS accumulation in aortas. Purslane extract improved endothelium‐dependent relaxations (EDRs) in ApoE^−/−^ mouse aortas and LPC‐treated mouse aortas. Protein expression analysis indicated that purslane extract suppressed vascular inflammation by suppressing the MAPK pathway and enhanced vasorelaxation by activating the AMPK/PI3K/Akt/eNOS pathway. These findings suggest that purslane extract is promising for inhibiting vascular inflammation and retarding the progression of atherosclerosis.

## Graphical abstract

Oral administration of purslane extract at 400 mg/lg/day for 4 weeks reduces atherosclerotic plaque formation, endothelial dysfunction, and vascular inflammation through suppressing the MAPK pathway and activating AMPK/PI3K/Akt/eNOS pathway in ApoE^−/−^ mice.

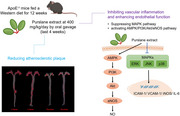

## 1 Introduction

Atherosclerosis is a chronic inflammatory disease occurring in the middle and large arteries [1]. An elevated level of plasma cholesterol is a clear risk factor for atherosclerosis [2]. A variety of inflammatory pathways and cytokines are involved in the pathogenesis of atherosclerosis [3]. Multiple systemic inflammation‐related proteins can serve as early biomarkers of atherosclerosis [4]. Endothelial dysfunction is the earliest change in the development of atherosclerosis, and endothelial cells are stimulated by a variety of lipoproteins and cytokines, leading to the formation of a pro‐inflammatory endothelial phenotype that initiates inflammation [5].

Endothelial dysfunction in the early stages of atherosclerosis is characterized by reduced bioavailability of nitric oxide (NO) produced by endothelial nitric oxide synthase (eNOS). Oxidized low‐density lipoprotein (oxLDL), a key atherogenic lipid, can decrease the expression of eNOS in endothelial cells [6]. Increased endogenous release of NO by eNOS has been shown to slow down atherosclerosis, associated with the reduction of oxidative stress and inflammation [7].

The mitogen‐activated protein kinase (MAPK) pathway is a triple‐kinase cascade that mediates cellular response to external stimuli, including inflammatory signals. Previous studies indicate that the MAPK pathway affects atherosclerosis. Stimulation with oxLDL can activate the MAPK pathway, resulting in significant increases in the phosphorylation of p38, c‐Jun N‐terminal kinase (JNK), and extracellular signal‐regulated kinase (ERK) in endothelial cells [8]. MAPK‐activated protein kinase 2 (MK2) is a direct substrate of p38, and its phosphorylation is highly expressed at atherosclerosis sites. Studies in MK2‐deficient LDL^−/−^ mice have demonstrated a significant inhibition of atherosclerosis [9].

*Portulaca oleracea* L. (purslane) is a widely distributed annual herb, valued for both medicinal and culinary purposes due to its rich nutrient profile. Purslane is documented in the “Compendium of Materia Medica” for its properties in dissipating blood stasis, eliminating swelling, detoxifying, and promoting diuresis. Purslane contains various bioactive components, including flavonoids, alkaloids, polysaccharides, and lignans [10]. Modern pharmacological studies have shown that the crude extract of purslane and its components exhibit significant anti‐inflammatory and antioxidant effects [11]. In addition, purslane shows regulatory effects on metabolic diseases such as diabetes, hypertension, and hyperlipidemia [12]. However, the effect of purslane on atherosclerosis, which is linked to several metabolic disorders, remains unclear.

Apolipoprotein E (ApoE) is a polymorphic protein involved in lipoprotein transformation and metabolism. ApoE knockout (ApoE^−/−^) mice have significantly higher total cholesterol levels compared to normal mice, which further increase up to four times when fed a Western diet [13]. This propensity leads to the rapid development of atherosclerotic plaques in ApoE^−/−^ mice, making them a well‐established model in atherosclerosis research. This study aims to investigate whether purslane extract can ameliorate atherosclerosis and protect against vascular inflammation in an ApoE^−/−^ mice model.

## 2 Material and Methods

### 2.1 Preparation of Purslane Extract

The shoot system of *P. oleracea* was collected in Anhui province, China. The plant material was air‐dried and then ground using a mill (Lingsum, China). The purslane powder was subjected to ultrasonic‐assisted extraction three times with 50% ethanol (w/v, 1:10) for 30 min per extraction. The combined extracts were filtered and concentrated under reduced pressure using a rotary evaporator. The concentrated liquid was freeze‐dried (Te Virtis Company, New York, NY, USA) to obtain the extract powder. The chemical profile of the purslane extract was analyzed by ultra‐performance liquid chromatography (UPLC) using a Waters Acquity UPLC BEH C18 Column. The mobile phase consisted of 0.2% H_3_PO_4_ in water and 0.2% H_3_PO_4_ in acetonitrile, with UV detection at 320 nm. A mixed standard solution containing five reference compounds, quercetin‐3‐O‐beta‐D‐glucose‐7‐O‐beta‐D‐gentiobioside, rutin, luteoloside, kaempferol‐3‐O‐rutinoside, and isochlorogenic acid A, was used for absolute quantification.

### 2.2 Animal Protocols

All animal experiments were approved by the Animal Research Ethics Committee, University of Macau (approved protocol no.: UMARE‐015‐2022) and the Guide for the Care and Use of Laboratory Animals. ApoE^−/−^ mice, aged 3 months, were fed a Western diet for 3 months. After 8 weeks on this diet, the mice were randomly divided into two groups (*n* = 14 per group). The treatment group received a daily oral gavage of purslane extract (400 mg/kg body weight) for the following 4 weeks, while the vehicle control group received an equivalent volume of ddH_2_O. Throughout this period, all mice continued to be fed the Western diet. The mice were housed in a controlled environment at a constant temperature of 22–23°C with a 12‐h light/12‐h dark cycle, and they had access to water ad libitum. The body weights of the mice were measured weekly. At the endpoint of the study, mice were euthanized by CO_2_ inhalation, and blood and aortic tissues were collected for subsequent analysis.

### 2.3 Determination of Plasma Lipid Profile and Inflammatory Cytokine Interleukin‐6 (IL‐6)

Following euthanasia by CO_2_, blood was collected from the inferior vena cava into heparin‐coated microcentrifuge tubes. Plasma was obtained by centrifuging the blood at 3000 rpm and 4°C for 10 min. Plasma lipid levels, including total cholesterol (TC), triglycerides (TG), high‐density lipoprotein cholesterol (HDL‐C), and low‐density lipoprotein cholesterol (LDL‐C), were quantified using commercial kits (Nanjing Jiancheng Bioengineering Institute, Nanjing, China). The concentration of IL‐6 was measured using a commercial ELISA kit according to the manufacturer's instructions (Neobioscience Bioengineering Institute, Shenzhen, China).

### 2.4 Oil red O Staining

Aortas were dissected from the aortic root to the iliac bifurcation, opened longitudinally, and fixed in 4% paraformaldehyde at 4°C for 16 h. After fixation, the aortas were washed with ddH_2_O for 10 min, followed by a rinse with 60% isopropanol for 2 min. Tissues were then stained with 0.5% Oil red O solution (in isopropanol) for 15 min. Excess stain was removed by washing with water and isopropanol. The stained aortas were photographed against a black background for analysis.

### 2.5 Detection of ROS by Dihydroethidium (DHE) Staining

Aortic segments were embedded in O.C.T compound and cryosectioned into 10 µm thick slices. The sections were incubated with 5 µM DHE solution dissolved in NPSS at 37°C in the dark for 30 min. Fluorescence images were captured using a Leica DMI8 inverted fluorescence microscope. Fluorescence intensity was quantified using Image J software (National Institute of Health, Bethesda, MA, USA).

### 2.6 Isometric force Measurement by Wire Myograph

Aortic segments (approx. 2 mm in length) from ApoE^−/−^ and C57BL/6J mice were mounted in a Multi Myograph System (Danish Myo Technology, Denmark) to measure isometric tension. Vessels were normalized to a baseline tension of 3 mN and equilibrated in Krebs solution (119 mM NaCl, 4.7 mM KCl, 2.5 mM CaCl_2_, 1 mM MgCl_2_, 25 mM NaHCO_3_, 1.2 mM KH_2_PO_4_, and 11 mM glucose, pH 7.4) for 60 min.

Vasoconstriction was induced with 60 mM KCl. Rings were then pre‐contracted with 3 µM phenylephrine (Phe, Sigma‐Aldrich, St. Louis, MO, USA). Endothelium‐dependent relaxation (EDR) was studied by cumulative addition of acetylcholine (ACh, Sigma–Aldrich, St. Louis, MO, USA) at concentrations ranging from 3 nM to 10 µM. In some experiments, Nω‐Nitro‐L‐arginine methyl ester hydrochloride (L‐NAME, Sigma–Aldrich, St. Louis, MO, USA) was utilized to inhibit nitric oxide synthase. Endothelium‐independent relaxations were evaluated by cumulative addition of sodium nitroprusside (SNP, Sigma–Aldrich, St. Louis, MO, USA) at concentrations ranging from 1 nM to 10 µM in the Phe‐precontracted rings.

### 2.7 Ex vivo Culture of Mouse Aortas

Aortas from C57BL/6J mice were dissected under sterile conditions and cultured in low‐glucose Dulbecco's modified Eagle's medium (DMEM, Gibco, Carlsbad, CA, USA) supplemented with 10% fetal bovine serum (FBS, Gibco, Carlsbad, CA, USA) and 1% penicillin/streptomycin (Gibco, Carlsbad, CA, USA) at 37°C in a 5% CO_2_ atmosphere.

Aortic segments were treated with 30 µM lysophosphatidylcholine (LPC, Sigma–Aldrich, St. Louis, MO, USA), either alone or co‐treated with purslane extract (100 or 400 µg/mL), at 37°C for 16 h. The control group received an equivalent volume of vehicle (water).

### 2.8 Western Blotting

Aortas from treated ApoE^−/−^ mice were homogenized on ice with RIPA lysis buffer (Beyotime Biotechnology, Shanghai, China) containing complete protease inhibitor cocktail (Roche, Basel, Switzerland) and PhosSTOP phosphatase inhibitors (Roche, Basel, Switzerland). Lysates were centrifuged at 150,000 rpm at 4°C for 30 min, and the supernatant was collected. Protein concentration was measured using the bicinchoninic acid (BCA) assay (Beyotime Biotechnology, Shanghai, China).

Protein samples (15 µg) were separated by 10% sodium dodecyl sulfate polyacrylamide gel electrophoresis (SDS‐PAGE) and transferred to PVDF membranes (Millipore, Billerica, MA, USA) via wet transfer. After blocking with 1% BSA in PBST, membranes were incubated overnight at 4°C with primary antibodies against: ICAM‐1, VCAM‐1, iNOS, PI3K, p‐AMPKα (Thr172), total AMPKα, p‐Akt (Ser473), total Akt, p‐eNOS (Ser1177), total eNOS, p‐JNK (Thr183/Tyr185), total JNK, p‐ERK1/2 (Thr202/Tyr204), total ERK, p‐p38 (Thr180/Tyr182), total p38, and GAPDH (all from Cell Signaling Technology, Danvers, MA, USA). Membranes were then incubated with the corresponding HRP‐coupled secondary antibody (Beyotime Biotechnology, Shanghai, China) at room temperature for 2 h. The antibody incubation method was determined based on the recommended method of the reagent supplier and with reference to the study of Luo et al. [14]. Protein bands were visualized using a chemiluminescent substrate (Thermo Fisher Scientific Inc., Waltham, MA, USA) and imaged with a ChemiDoc^TM^ MP Imaging System (BIO‐RAD, Hercules, CA, USA). Band intensity was analyzed by ImageJ software (National Institute of Health, Bethesda, MA, USA).

### 2.9 Statistical Analysis

Data are presented as the mean ± standard error of mean (SEM) from *n*‐independent experiments. Statistical comparisons were performed using GraphPad Prism software (GraphPad Software, San Diego, CA, USA). Differences between groups were analyzed by Student's *t*‐test or one‐way ANOVA followed by Bonferroni's post hoc test, as appropriate. A *p*‐value of less than 0.05 was considered to indicate a statistically significant difference.

## 3 Results

### 3.1 The Main Components in Purslane Extract

**FIGURE 1:**
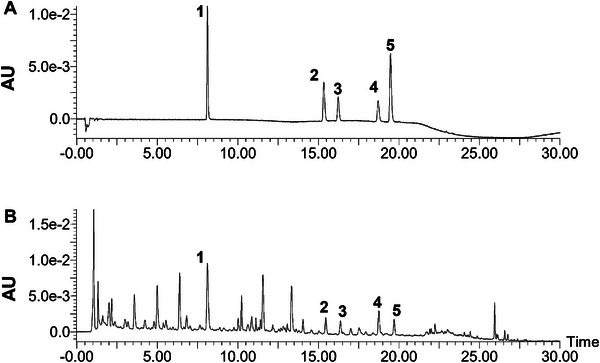
The UPLC profiles of (A) mixed standards and (B) purslane extract. 1: Quercetin‐3‐O‐beta‐D‐glucose‐7‐O‐beta‐D‐gentiobioside; 2: Rutin; 3: Luteoloside; 4: Kaempferol‐3‐O‐rutinoside; 5: Isochlorogenic acid A.

The extraction of 16.5 g of dried *P. oleracea* plant material yielded 2.54 g of extract powder, corresponding to an extraction yield of 15.4%. Five compounds were quantified in purslane extract by comparison with standard substances, focusing on flavonoids and phenolic acids known for their pharmacological activities. The contents of the five compounds detected, expressed as a percentage of the extract mass, were: quercetin‐3‐O‐beta‐D‐glucose‐7‐O‐beta‐D‐gentiobioside (0.103%), rutin (0.018%), luteoloside (0.010%), kaempferol‐3‐O‐rutinoside (0.020%), and isochlorogenic acid A (0.004%) (Figure 1).

### 3.2 Purslane Extract Ameliorates Dyslipidemia and Reduces Plasma IL‐6 in ApoE^−/−^ Mice

**FIGURE 2:**
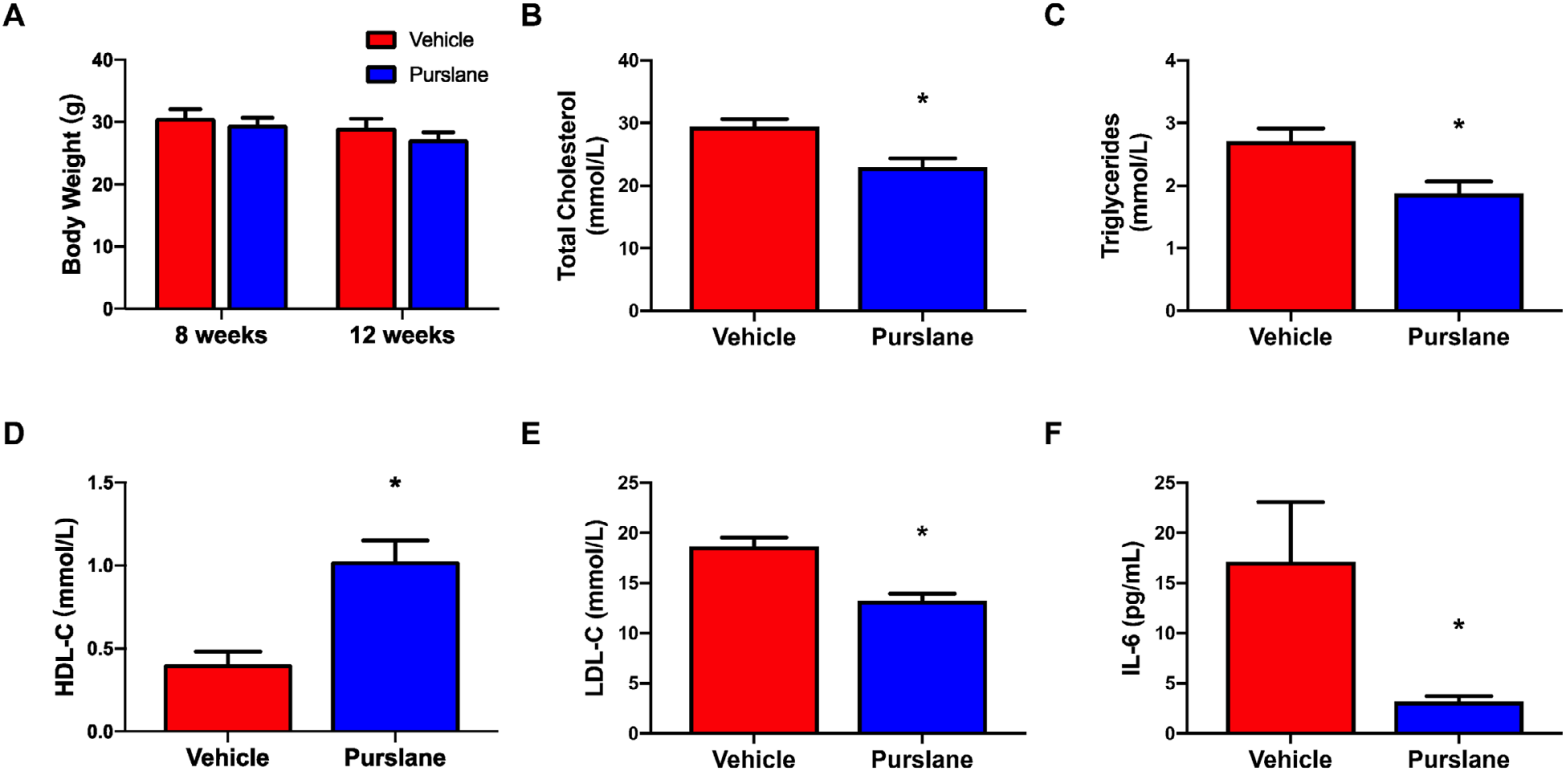
Effects of purslane extract on body weight, plasma lipid, and plasma inflammatory cytokine in Western diet‐induced ApoE^−/−^ mice. (A) Body weight of ApoE^−/−^ mice before and after oral administration of vehicle and purslane (Vehicle vs. Purslane, 8 weeks: *p *= 0.5340; 12 weeks: *p *= 0.2823). Levels of (B) total cholesterol (*p* = 0.0018), (C) triglycerides (*p* = 0.0056), (D) HDL‐C (*p* = 0.0004), (E) LDL‐C (*p* < 0.0001), and (F) interleukin‐6 (IL‐6) (*p* = 0.0289). Data are mean ± SEM, *n* = 13∼14, biological replicates. ∗*p* < 0.05 Vehicle versus Purslane, *t*‐test (unpaired).

Four‐week purslane extract administration had no significant effect on body weight (8 weeks: *p* = 0.5340; 12 weeks: *p* = 0.2823) (Figure 2A). The purslane extract treatment significantly reduced plasma levels of TC (*p* = 0.0018), TG (*p* = 0.0056), and LDL‐C (*p* *<* 0.0001), whereas it increased HDL‐C (*p* = 0.0004) in Western diet‐induced ApoE^−/−^ mice (Figure 2B–E). Furthermore, the plasma concentration of the pro‐inflammatory cytokine IL‐6 was also significantly reduced following treatment (*p* = 0.0289) (Figure 2F). These results demonstrate that purslane extract effectively improves lipid profiles and reduces systemic inflammation in Western diet‐fed ApoE^−/−^ mice.

### 3.3 Purslane Extract Reduces Atherosclerotic Plaque Formation, Inhibits ROS Accumulation, and Improves Endothelial Function in ApoE^−/−^ Mice

**FIGURE 3:**
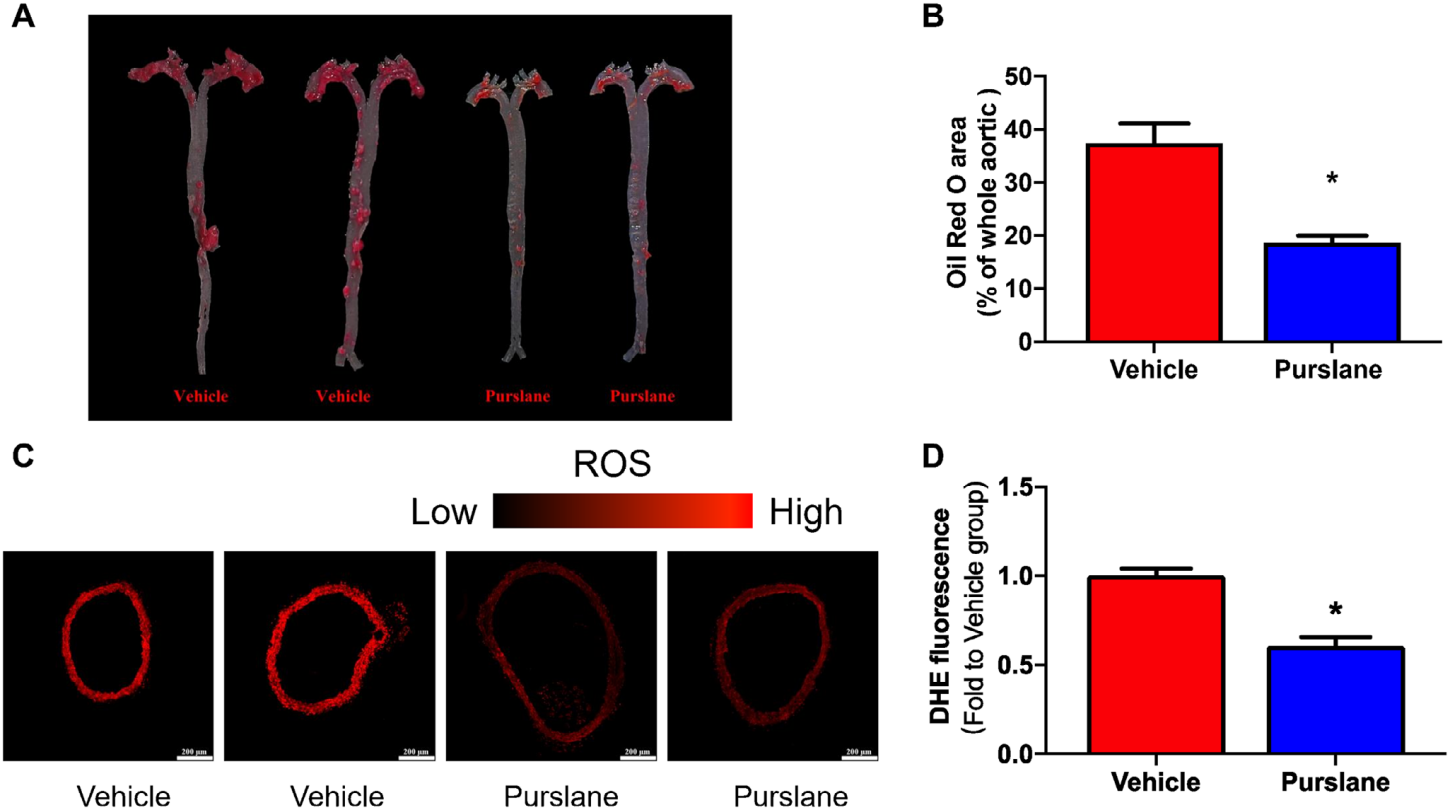
Purslane extract ameliorates atherosclerotic plaque formation and oxidative stress in ApoE^−/−^ mice. (A) Representative images of aortas with Oil red O staining and (B) ratio of the area of atherosclerotic plaque to the area of the entire aorta (*p* = 0.0005). (C) Respective images (scale bar: 200 µm) and (D) statistical graph of DHE intensity (ROS release) in aortas from ApoE^−/−^ mice (*p* < 0.0001). Data are mean ± SEM, *n* = 7, biological replicates. ∗*p* < 0.05 Vehicle versus Purslane, *t*‐test (unpaired).

After 12 weeks on a Western diet, the vehicle‐treated ApoE^−/−^ mice developed severe atherosclerosis, characterized by the accumulation of plaques primarily in the aortic arch and blood vessel branches. Remarkably, treatment with purslane extract for 4 weeks resulted in a significant alleviation of plaque formation (*p* = 0.0005) (Figure 3A,B). This result demonstrates the efficacy of purslane extract in mitigating the progression of atherosclerosis.

The occurrence and development of atherosclerosis are closely related to oxidative stress. A four‐week course of orally administered purslane extract significantly diminished ROS accumulation in the aortas of ApoE^−/−^ mice (*p* < 0.0001) (Figure 3C,D).

**FIGURE 4:**
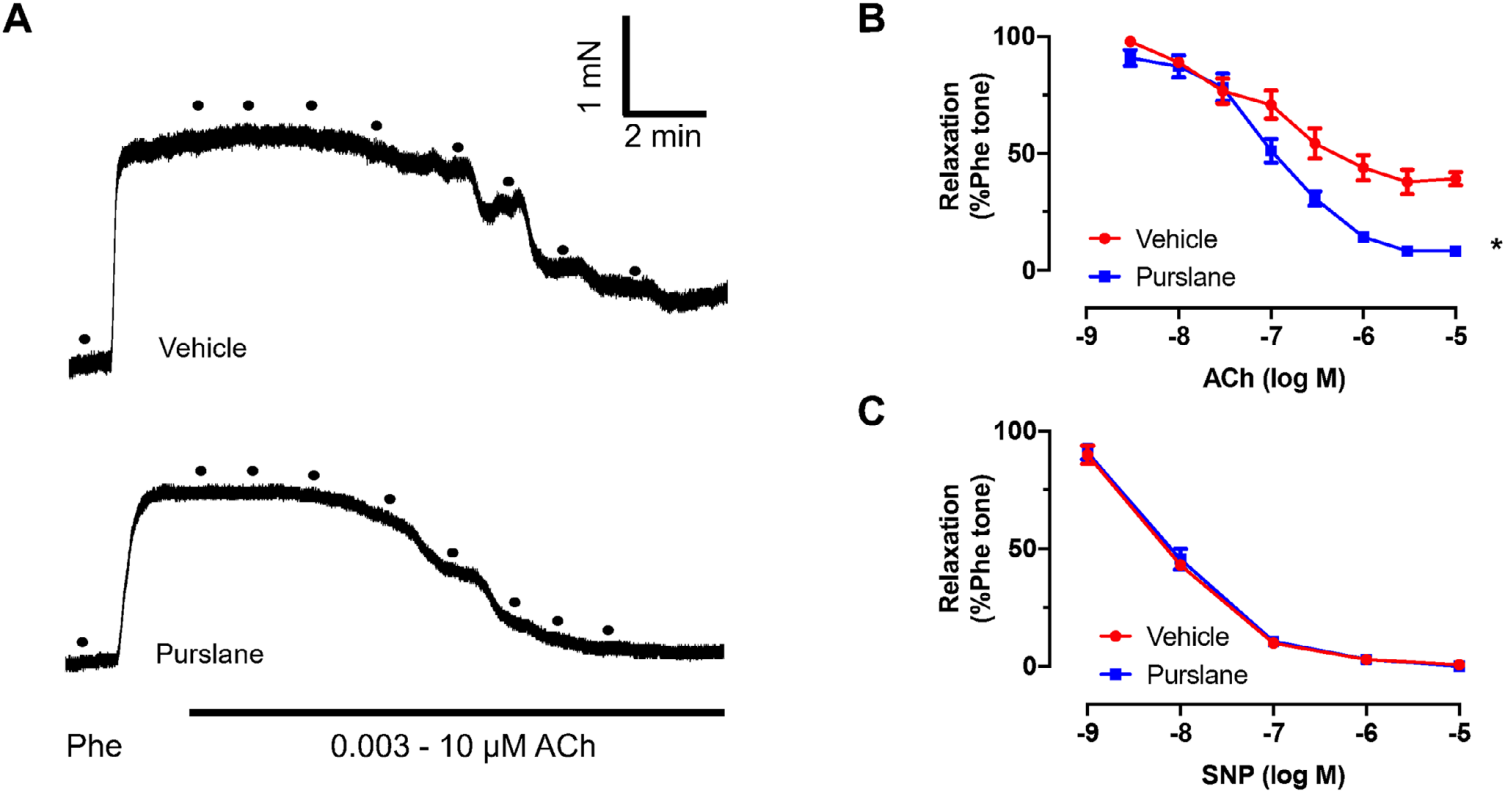
Purslane extract ameliorates vasorelaxations in aortas of ApoE^−/−^ mice with atherosclerosis. (A) Representative traces and (B) summarized graph showing effects of oral administration of purslane (400 mg/kg/day for 4 weeks) on acetylcholine (ACh)‐induced EDRs in the aortas from ApoE^−/−^ mice (*p*<0.0001). (C) Sodium nitroprusside (SNP)‐induced endothelium‐independent relaxations in the aortas of ApoE^−/−^ mice (*p* = 0.6133). Data are mean ± SEM, *n* = 7, biological replicates. ∗*p* < 0.05 Vehicle versus Purslane, *t*‐test (unpaired).

After 3 months on a Western diet, acetylcholine (ACh)‐induced EDRs were impaired in ApoE^−/−^ mice. Four weeks of oral treatment with purslane extract significantly improved EDR compared to untreated controls (*p* < 0.0001) (Figure 4A,B). In contrast, sodium nitroprusside (SNP)‐induced endothelium‐independent relaxation was not significantly different between the groups (*p* = 0.6133) (Figure 4C).

### 3.4 Purslane Extract Suppresses Inflammation in Aortas From ApoE^−/−^ Mice

**FIGURE 5:**
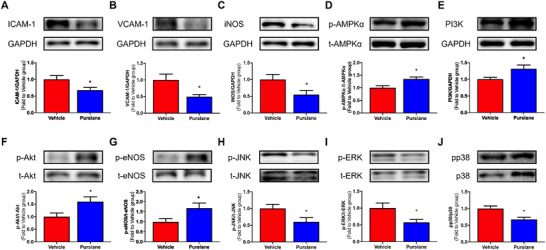
Effect of purslane extract on AMPK/Akt/eNOS and MAPK signaling pathways in the ApoE^−/−^ mouse aortas. Representative blots and summarized data showing the effect of purslane extract treatment (400 mg/kg body weight, 4 weeks) on protein expressions of (A) ICAM‐1 (90 kDa) (*p* = 0.0408), (B) VCAM‐1 (110 kDa) (*p* = 0.0206) and (C) iNOS (130 kDa) (*p* = 0.0443) compared to GAPDH (36 kDa); (D) phosphorylation of AMPKα at Thr172 (p‐AMPKα; 62 kDa) compared to its total protein (t‐AMPKα) (*p* = 0.0105); (E) PI3 kinase (PI3K; 85 kDa) compared to GAPDH (36 kDa) (*p* = 0.0339); (F) phosphorylation of Akt at Ser473 (p‐Akt; 60 kDa) compared to its total protein (t‐Akt) (*p* = 0.0364); (G) phosphorylation of eNOS at Ser1177 (p‐eNOS; 140 kDa) compared to its total protein (t‐eNOS) (*p* = 0.0455); (H) phosphorylation of JNK at Thr183/Tyr185 (p‐JNK; 46/54 kDa) compared to its total protein (t‐JNK) (*p* = 0.0495); (I) phosphorylation of ERK(1/2) at Thr202/Tyr204 (p‐ERK; 42/44 kDa) compared to its total protein (t‐ERK) (*p* = 0.0328); and (J) phosphorylation of p38 MAPK at Thr180/Tyr182 (pp38; 43 kDa) compared to its total protein (t‐p38) (*p* = 0.0071). Data are mean ± SEM, *n* = 6∼7, biological replicates. ∗*p* < 0.05 Vehicle versus Purslane, *t*‐test (unpaired).

To investigate the mechanisms underlying the vascular benefits of purslane extract, protein expressions related to inflammatory and NO pathways in aortic homogenates from ApoE^−/−^ mice were measured. Four weeks of purslane treatment significantly suppressed the expression of the inflammatory markers ICAM‐1 (*p* = 0.0408), VCAM‐1 (*p* = 0.0206), and iNOS (*p* = 0.0443) (Figure 5A,C). Furthermore, the extract upregulated the expression of PI3K (*p* = 0.0339) and enhanced the phosphorylation of AMPKα at Thr172 (*p* = 0.0105), Akt at Ser473 (*p* = 0.0364), and eNOS at Ser1177 (*p* = 0.0455) (Figure 5D‐G). On the other hand, purslane extract significantly inhibited the phosphorylation of JNK at Thr183/Tyr185 (*p* = 0.0495), ERK at Thr202/Tyr204 (*p* = 0.0328), and p38 at Thr180/Tyr182 (*p* = 0.0071) in the aortas of the treated mice (Figure 5H‐J). The molecular changes, promoting the PI3K/Akt/eNOS pathway while suppressing inflammatory and MAPK signaling, are consistent with the observed improvement in endothelial function.

### 3.5 Purslane Extract Ameliorates LPC‐Induced Endothelial Dysfunction Ex Vivo

**FIGURE 6:**
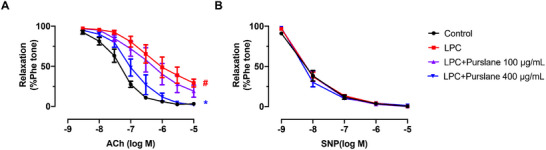
Vasoprotective effect of purslane extract in aortas from C57BL/6J mice ex vivo. Effect of purslane extract (100 and 400 µg/mL) on (A) acetylcholine (ACh)‐induced EDRs of aortas (Control vs. LPC, *p* < 0.0001; LPC vs. LPC+Purslane 100 µg/mL, *p* = 0.2239; LPC vs. LPC+Purslane 400 µg/mL, *p* < 0.0001) and (B) sodium nitroprusside (SNP)‐induced endothelium‐independent relaxations of aortas (Control vs. LPC, *p* = 0.8818; LPC vs. LPC+Purslane 100 µg/mL, *p* = 0.9857; LPC vs. LPC+Purslane 400 µg/mL, *p* = 0.8232) from mice exposed to lysophosphatidylcholine (LPC, 30 mM, 16 h). Data are mean ± SEM, *n* = 5, biological replicates. # *p* < 0.05 LPC versus NC; * *p* < 0.05 LPC + Purslane versus LPC, one‐way ANOVA followed by Bonferroni's post hoc test.

The protective effect of purslane extract was further validated using an ex vivo model of endothelial dysfunction. In this model, aortic segments from C57BL/6J mice were cultured with 30 µM LPC, a major component of oxLDL, for 16 h to simulate a pro‐atherosclerotic environment. As expected, LPC significantly impaired ACh‐induced EDR (*p* < 0.0001). Co‐incubation with purslane extract at 100 µg/mL did not rescue this impairment (*p* = 0.2239). However, a higher concentration of 400 µg/mL p significantly ameliorated the LPC‐impaired EDR (*p* < 0.0001) (Figure 6A). In contrast, SNP‐induced endothelium‐independent relaxation was unaffected by either LPC or purslane treatment (Figure 6B).

## 4 Discussion

Atherosclerosis is driven by interrelated disturbances in lipid metabolism, oxidative stress, and inflammation. Previous studies have reported the antioxidant and anti‐inflammatory properties of purslane extract in various models. Purslane extract mitigates oxidative stress by reducing markers like malondialdehyde (MDA) and myeloperoxidase (MPO) in lipopolysaccharide (LPS)‐induced lung injury [15] and protects against dexamethasone‐induced impairment of sperm production and motility [16]. It also plays an important role in improving solar dermatitis [17]. Its anti‐inflammatory efficacy is evidenced by the ability of a hydroethanolic extract to reduce postoperative peritoneal adhesion [18], and of both water and ethanol extracts to ameliorate colitis in mice by suppressing inflammatory cytokines and tissue damage [19, 20]. Purslane extraction also alleviates intestinal inflammation in obese mice [21]. These findings align with the antioxidant and anti‐inflammatory activities of purslane observed in aortas from ApoE^−/−^ mice.

Notably, prior research has demonstrated the significant effect of purslane in improving vascular injury and lipid metabolism. Purslane water extract inhibits tumor necrosis factor alpha (TNF‐α)‐induced NF‐κB activation in human umbilical vein endothelial cells (HUVECs) [22], and its extract suppresses inflammation in LPS‐induced macrophages by inhibiting NF‐κB and MAPK signaling pathways [23]. Moreover, purslane ethanol extract improves lipid metabolism and reduces serum inflammatory cytokines [24]. Similarly, its water extract ameliorates diabetic parameters and vascular dysfunction in *db/db* mice, concomitantly reducing the aortic expression of ICAM‐1, VCAM‐1, and endothelin‐1 (ET‐1) [25]. Our results confirm and extend these observations by showing that 50% ethanol‐water extract of purslane confers anti‐atherogenic effects and exhibits potent anti‐inflammatory properties and lipid‐regulating activities.

Vascular inflammation in atherosclerosis is driven by the upregulation of cell adhesion molecules such as ICAM‐1 and VCAM‐1, which mediate leukocyte adhesion to the endothelium. These molecules are highly expressed in the atherosclerosis‐prone sites of ApoE^−/−^ mice [26, 27], and therapeutic strategies targeting them, particularly VCAM‐1, show promise for treating atherosclerosis [28]. Within endothelial cells, iNOS promotes the expression of these adhesion factors, and its deficiency can reverse TNF‐α‐induced endothelial dysfunction [29].

The MAPK pathway is a recognized target for anti‐inflammation interventions. Clinical trials have demonstrated that p38 inhibition reduces vascular inflammation in atherosclerotic patients on statin therapy [30]. Exercise has also been shown to ameliorate atherosclerosis and reduce vascular inflammation in ApoE^−/−^ mice, concomitant with downregulation of ERK1/2 and MEK1/2 phosphorylation [31]. These studies show that intervening in the MAPK inflammatory pathway can alleviate atherosclerosis. The current study indicate that purslane extract alleviates vascular inflammation by suppressing the MAPK pathway, thereby retarding the progression of atherosclerosis.

Endothelial dysfunction is a reversible early stage in atherogenesis [32]. A previous study has demonstrated that purslane extract reverses endothelial dysfunction in diabetes and obesity [33]. Likewise, the present study shows that purslane treatment effectively restores vasorelaxation in atherosclerotic mice. The direct vascular action of purslane was further confirmed ex vivo, using LPC, a major component of oxidized LDL‐C to induce endothelial dysfunction in mouse aortas [34]. While the normalization of the plasma lipid profile, especially the reduction in LDL‐C and triglycerides, undoubtedly contributes to the anti‐atherogenic effect [35, 36], the ex vivo data demonstrate that purslane also confers direct, lipid‐independent protection to the vasculature.

Reduced eNOS expression and NO release are characteristic of atherosclerotic aortas [37], and targeted eNOS delivery has been shown to reduce atherosclerotic plaque formation in LDLR^−/−^ mice, highlighting eNOS activation as a promising therapeutic strategy [38]. The PI3K/Akt pathway is a key positive regulator of eNOS; PI3K activates Akt, which subsequently phosphorylates eNOS at Ser1177 to stimulate NO production [39]. Besides, AMP‐activated protein kinase (AMPK) also promotes eNOS activation. AMPK activator AICAR stimulates eNOS phosphorylation at Ser1177 and promotes NO production in human aortic endothelial cells [40]. In vivo, high‐fat diets downregulate the AMPK and PI3K/Akt/eNOS pathways, reducing NO availability and inducing endothelial dysfunction. The dependence of this signaling cascade is demonstrated by the results that inhibitors of PI3K (wortmannin), Akt (triciribine), and eNOS (L‐NAME) all attenuate AICAR‐induced vasorelaxation [41]. Collectively, these findings suggest that AMPK enhances vascular NO availability via the PI3K/Akt/eNOS pathway. The present data indicate that purslane extract activates AMPK/PI3K/Akt/eNOS axis, thereby improving endothelium‐dependent vasorelaxation.

*P. oleracea*. is rich in bioactive flavonoids and phenolic acids [42, 43], which are likely responsible for its pharmacological activities. As the plant source, plant parts, extraction solvent system, and method all have an impact on the composition and content of the compounds in the purslane extract. Previous studies show that the most abundant flavonoids include catechin, quercetin, kaempferol, and rutin, while the abundant phenolic acids are caffeic acid and chlorogenic acid [42, 43, 44, 45, 46]. Among them, some major flavonoids and the glycosides as well as one phenolic acid were qualitatively and quantitively identified, including quercetin‐3‐O‐beta‐D‐glucose‐7‐O‐beta‐D‐gentiobioside (QGG), kaempferol‐3‐O‐rutinoside, rutin, luteoloside, and isochlorogenic acid A.

These compounds exhibit mechanism that align closely with our findings. Specifically, QGG demonstrates lipid‐lowering [47] and antioxidant effects via the Akt pathway [48]. Rutin modulates lipid metabolism through AMPK [49] and protects the vascular endothelium by inhibiting NF‐κB and CAM expression [50], in addition to its antioxidant activity via the Nrf2 pathway [51]. Similarly, kaempferol‐3‐O‐rutinoside exhibits anti‐inflammatory properties by inhibiting both the NF‐κB [52] and MAPK pathways [53], as well as hepatoprotective effects through the reduction of oxidative stress [54]. Furthermore, luteoloside demonstrates a multifaceted mechanism, exerting anti‐inflammatory effects via the TLR4/ NF‐κB and P2X7R/NLRP3 pathway and antioxidant effects via the Nrf2/HO‐1 axis, which collaboratively mitigate tissue injury [55, 56]. Future studies are warranted to definitively establish the contribution of these individual bioactive compounds to the observed benefits of purslane extract against atherosclerosis.

## 5 Conclusion

In conclusion, chronic administration of purslane extract demonstrates a significantly mitigates Western diet‐induced atherosclerosis in ApoE^−/−^ mice. The atheroprotective effects are mediated by the amelioration of endothelial dysfunction, reduction of vascular inflammation and oxidative stress, and normalization of the plasma lipid profile. At the molecular level, purslane extract suppressed MAPK signaling and activated AMPK/PI3K/Akt/eNOS pathway. These findings highlight purslane's promising potential as a therapeutic agent or nutraceutical against atherosclerosis.

## Funding

This work was supported by the Science and Technology Development Fund, Macau SAR (0002/2025/NRP), University of Macau and the University of Macau Development Foundation (MYRG‐GRG2023‐00212‐ICMS‐UMDF and MYRG‐GRG2024‐00258‐ICMS‐UMDF), and Young Talent Support Project of Guangzhou Association for Science and Technology (QT2024‐048).

## Ethics Statement

The animal study protocol was approved by the Animal Research Ethics Committee, University of Macau (protocol no.: UMARE‐015‐2022 approved on July 26, 2022).

## Consent

The authors have nothing to report.

## Conflicts of Interest

The authors declare no conflicts of interest.

## Data Availability

The data that support the findings of this study are available from the corresponding author upon reasonable request.

## References

[1] K. J. Moore , F. J. Sheedy , and E. A. Fisher , “Macrophages in Atherosclerosis: A Dynamic Balance,” Nature Reviews Immunology 13 (2013): 709–721, 10.1038/nri3520. PMC4357520 23995626

[2] E. Falk , “Pathogenesis of Atherosclerosis,” Journal of the American College of Cardiology 47 (2006): C7–C12, 10.1016/j.jacc.2005.09.068. 16631513

[3] P. Kong , Z.‐Y. Cui , X.‐F. Huang , D.‐D. Zhang , R.‐J. Guo , and M. Han , “Inflammation and Atherosclerosis: Signaling Pathways and Therapeutic Intervention,” Signal Transduction and Targeted Therapy 7 (2022): 131, 10.1038/s41392-022-00955-7. 35459215 PMC9033871

[4] D. J. Medina‐Leyte , O. Zepeda‐García , M. Domínguez‐Pérez , A. González‐Garrido , T. Villarreal‐Molina , and L. Jacobo‐Albavera , “Endothelial Dysfunction, Inflammation and Coronary Artery Disease: Potential Biomarkers and Promising Therapeutical Approaches,” International Journal of Molecular Sciences 22 (2021): 3850, 10.3390/ijms22083850. 33917744 PMC8068178

[5] M. A. Gimbrone and G. García‐Cardeña , “Endothelial Cell Dysfunction and the Pathobiology of Atherosclerosis,” Circulation Research 118 (2016): 620–636, 10.1161/CIRCRESAHA.115.306301. 26892962 PMC4762052

[6] T. Bai , M. Li , Y. Liu , Z. Qiao , and Z. Wang , “Inhibition of Ferroptosis Alleviates Atherosclerosis Through Attenuating Lipid Peroxidation and Endothelial Dysfunction in Mouse Aortic Endothelial Cell,” Free Radical Biology and Medicine 160 (2020): 92–102, 10.1016/j.freeradbiomed.2020.07.026. 32768568

[7] A. Sharma , S. Sellers , N. Stefanovic , et al., “Direct Endothelial Nitric Oxide Synthase Activation Provides Atheroprotection in Diabetes‐Accelerated Atherosclerosis,” Diabetes 64 (2015): 3937–3950, 10.2337/db15-0472. 26116699

[8] Y. Yin , W. Liu , G. Ji , and Y. Dai , “The Essential Role of p38 MAPK in Mediating the Interplay of oxLDL and IL‐10 in Regulating Endothelial Cell Apoptosis,” European Journal of Cell Biology 92 (2013): 150–159, 10.1016/j.ejcb.2013.01.001. 23498167

[9] K. Jagavelu , U. J. F. Tietge , M. Gaestel , H. Drexler , B. Schieffer , and U. Bavendiek , “Systemic Deficiency of the MAP Kinase–Activated Protein Kinase 2 Reduces Atherosclerosis in Hypercholesterolemic Mice,” Circulation Research 101 (2007): 1104–1112, 10.1161/CIRCRESAHA.107.156075. 17885219

[10] Y. Li , L. Xiao , H. Yan , et al., “Nutritional Values, Bioactive Compounds and Health Benefits of purslane (*Portulaca oleracea* L): A Comprehensive Review,” Food Science and Human Wellness 13 (2024): 2480–2501.

[11] V. Ghorani , S. Saadat , M. R. Khazdair , et al., “Phytochemical Characteristics and Anti‐Inflammatory, Immunoregulatory, and Antioxidant Effects of *Portulaca Oleracea* L.: A Comprehensive Review,” Evidence‐Based Complementary and Alternative Medicine 2023, 2023, 2075444. 37693918 10.1155/2023/2075444 PMC10484659

[12] J. Jalali and M. Ghasemzadeh Rahbardar , “Ameliorative Effects of *Portulaca Oleracea* L. (purslane) on the Metabolic Syndrome: A Review,” Journal of Ethnopharmacology 299 (2022): 115672, 10.1016/j.jep.2022.115672. 36064150

[13] K. S. Meir and E. Leitersdorf , “Atherosclerosis in the Apolipoprotein E–Deficient Mouse,” Arteriosclerosis, Thrombosis, and Vascular Biology 2004, 24, 1006–1014. 15087308 10.1161/01.ATV.0000128849.12617.f4

[14] H. Luo , G. O. Rankin , S. Straley , and Y. C. Chen , “Prolonged Incubation and Stacked Film Exposure Improve Sensitivity in Western Blotting,” Journal of Pharmacological and Toxicological Methods 64 (2011): 233–237, 10.1016/j.vascn.2011.06.001. 21741488 PMC3204169

[15] V. Baradaran Rahimi , H. Rakhshandeh , F. Raucci , et al., “Anti‐Inflammatory and Anti‐Oxidant Activity of *Portulaca Oleracea* Extract on LPS‐Induced Rat Lung Injury,” Molecules 24 (2019): 139. 30609661 10.3390/molecules24010139 PMC6337267

[16] S. R. Saleh , A. Manaa , E. Sheta , D. A. Ghareeb , and N. M. Abd‐Elmonem , “The Synergetic Effect of Egyptian *Portulaca Oleracea* L. (Purslane) and Cichorium Intybus L. (Chicory) Extracts Against Glucocorticoid‐Induced Testicular Toxicity in Rats Through Attenuation of Oxidative Reactions and Autophagy,” Antioxidants 11 (2022): 1272. 35883763 10.3390/antiox11071272 PMC9311541

[17] B. Li , J. Yang , Z. Wang , et al., “Extracts of *Portulaca Oleracea* and Patrinia Scabiosaefolia Relieve Ultraviolet B‐Induced Skin Injury in Solar Dermatitis Mice via Inhibiting IL‐17/CCL2 Pathway and Oxidative Stress,” International Journal of Medical Sciences 22 (2025): 856–872, 10.7150/ijms.106289. 39991764 PMC11843150

[18] A. Jaafari , V. Baradaran Rahimi , N. Vahdati‐Mashhadian , et al., “Evaluation of the Therapeutic Effects of the Hydroethanolic Extract of *Portulaca Oleracea* on Surgical‐Induced Peritoneal Adhesion,” Mediators of Inflammation 2021 (2021): 8437753, 10.1155/2021/8437753. 34381307 PMC8352699

[19] Z. Zhang , D. Qiao , Y. Zhang , et al., “ *Portulaca Oleracea* L Extract Ameliorates Intestinal Inflammation by Regulating Endoplasmic Reticulum Stress and Autophagy,” Molecular Nutrition & Food Research 66 (2022): 2100791. 34968000 10.1002/mnfr.202100791 PMC9286603

[20] Y. Kim , H. J. Lim , H.‐J. Jang , et al., “ *Portulaca Oleracea* Extracts and Their Active Compounds Ameliorate Inflammatory Bowel Diseases In Vitro and In Vivo by Modulating TNF‐α, IL‐6 and IL‐1β Signalling,” Food Research International 106 (2018): 335–343, 10.1016/j.foodres.2017.12.058. 29579933

[21] L. Miao , M. S. Cheong , H. Zhang , et al., “ *Portulaca Oleracea* L. (purslane) Extract Ameliorates Intestinal Inflammation in Diet‐Induced Obese Mice by Inhibiting the TLR4/NF‐κB Signaling Pathway,” Frontiers in Pharmacology 15 (2024): 1474989, 10.3389/fphar.2024.1474989. 39845784 PMC11752911

[22] A. S. Lee , J. S. Kim , Y. J. Lee , D. G. Kang , and H. S. Lee , “Anti‐TNF‐α Activity of *Portulaca Oleracea* in Vascular Endothelial Cells,” International Journal of Molecular Sciences 13 (2012): 5628–5644, 10.3390/ijms13055628. 22754320 PMC3382818

[23] L. Miao , H. Tao , Y. Peng , et al., “The Anti‐Inflammatory Potential of *Portulaca Oleracea* L. (purslane) extract by Partial Suppression on NF‐κB and MAPK Activation,” Food Chemistry 290 (2019): 239–245, 10.1016/j.foodchem.2019.04.005. 31000042

[24] G. Zheng , F. Mo , C. Ling , et al., “ *Portulaca Oleracea* L. Alleviates Liver Injury in Streptozotocin‐Induced Diabetic Mice,” Drug Design, Development and Therapy 12 (2018): 47–55, 10.2147/DDDT.S121084. 29343942 PMC5749558

[25] A. S. Lee , Y. J. Lee , S. M. Lee , et al., “ *Portulaca Oleracea* Ameliorates Diabetic Vascular Inflammation and Endothelial Dysfunction in db/db Mice,” Evidence‐Based Complementary and Alternative Medicine 2012 (2012): 741824. 22474522 10.1155/2012/741824 PMC3303738

[26] Y. Nakashima , E. W. Raines , A. S. Plump , J. L. Breslow , and R. Ross , “Upregulation of VCAM‐1 and ICAM‐1 at Atherosclerosis‐Prone Sites on the Endothelium in the ApoE‐Deficient Mouse,” Arteriosclerosis, Thrombosis, and Vascular Biology 18 (1998): 842–851, 10.1161/01.ATV.18.5.842. 9598845

[27] V. Singh , R. Kaur , P. Kumari , C. Pasricha , and R. Singh , “ICAM‐1 and VCAM‐1: Gatekeepers in Various Inflammatory and Cardiovascular Disorders,” Clinica Chimica Acta 548 (2023): 117487, 10.1016/j.cca.2023.117487. 37442359

[28] J. R. Pickett , Y. Wu , L. F. Zacchi , and H. T. Ta , “Targeting Endothelial Vascular Cell Adhesion Molecule‐1 in Atherosclerosis: Drug Discovery and Development of Vascular Cell Adhesion Molecule‐1–Directed Novel Therapeutics,” Cardiovascular Research 119 (2023): 2278–2293, 10.1093/cvr/cvad130. 37595265 PMC10597632

[29] C. Liu , S. Lei , T. Cai , et al., “Inducible Nitric Oxide Synthase Activity Mediates TNF‐α‐Induced Endothelial Cell Dysfunction,” American Journal of Physiology‐Cell Physiology 325 (2023): C780–C795, 10.1152/ajpcell.00153.2023. 37575057

[30] M. Elkhawad , J. H. F. Rudd , L. Sarov‐Blat , et al., “Effects of p38 Mitogen‐Activated Protein Kinase Inhibition on Vascular and Systemic Inflammation in Patients with Atherosclerosis,” JACC: Cardiovascular Imaging 5 (2012): 911–922, 10.1016/j.jcmg.2012.02.016. 22974804

[31] Y. Wang , L. Chen , M. Zhang , et al., “Exercise‐Induced Endothelial Mecp2 Lactylation Suppresses Atherosclerosis via the Ereg/MAPK Signalling Pathway,” Atherosclerosis 375 (2023): 45–58, 10.1016/j.atherosclerosis.2023.05.009. 37245426

[32] S. Sitia , L. Tomasoni , F. Atzeni , et al., “From Endothelial Dysfunction to Atherosclerosis,” Autoimmunity Reviews 9 (2010): 830–834, 10.1016/j.autrev.2010.07.016. 20678595

[33] L. Miao , C. Zhou , H. Zhang , et al., “ *Portulaca Oleracea* L (Purslane) Extract Protects Endothelial Function by Reducing Endoplasmic Reticulum Stress and Oxidative Stress through AMPK Activation in Diabetic Obese Mice,” Antioxidants 12 (2023): 2132. 38136251 10.3390/antiox12122132 PMC10741183

[34] A. Pirillo , G. D. Norata , and A. L. Catapano , “LOX‐1, OxLDL, and Atherosclerosis,” Mediators of Inflammation 2013 (2013): 152786. 23935243 10.1155/2013/152786 PMC3723318

[35] P. B. Sandesara , S. S. Virani , S. Fazio , and M. D. Shapiro , “The Forgotten Lipids: Triglycerides, Remnant Cholesterol, and Atherosclerotic Cardiovascular Disease Risk,” Endocrine Reviews 40 (2018): 537–557, 10.1210/er.2018-00184. PMC6416708 30312399

[36] F. Mach , C. Baigent , A. L. Catapano , et al., “2019 ESC/EAS Guidelines for the Management of Dyslipidaemias: Lipid Modification to Reduce Cardiovascular Risk: European Society of Cardiology (ESC) and European Atherosclerosis Society (EAS),” European Heart Journal 41 (2019): 111–188, 10.1093/eurheartj/ehz455. 31504418

[37] B. S. Oemar , M. R. Tschudi , N. Godoy , V. Brovkovich , T. Malinski , and T. F. Lu?scher , “Reduced Endothelial Nitric Oxide Synthase Expression and Production in Human Atherosclerosis,” Circulation 97 (1998): 2494–2498, 10.1161/01.CIR.97.25.2494. 9657467

[38] Q. Ul Ain , H. Chung , J. Y. Chung , J.‐H. Choi , and Y.‐H. Kim , “Amelioration of Atherosclerotic Inflammation and Plaques via Endothelial Adrenoceptor‐Targeted eNOS Gene Delivery Using Redox‐Sensitive Polymer Bearing L‐Arginine,” Journal of Controlled Release 262 (2017): 72–86, 10.1016/j.jconrel.2017.07.019. 28710003

[39] F. Morello , A. Perino , and E. Hirsch , “Phosphoinositide 3‐Kinase Signalling in the Vascular System,” Cardiovascular Research 82 (2008): 261–271, 10.1093/cvr/cvn325. 19038971

[40] V. A. Morrow , F. Foufelle , J. M. C. Connell , J. R. Petrie , G. W. Gould , and I. P. Salt , “Direct Activation of AMP‐Activated Protein Kinase Stimulates Nitric‐Oxide Synthesis in Human Aortic Endothelial Cells,” Journal of Biological Chemistry 278 (2003): 31629–31639, 10.1074/jbc.M212831200. 12791703

[41] C. F. García‐Prieto , F. Hernández‐Nuño , D. D. Rio , et al., “High‐Fat Diet Induces Endothelial Dysfunction Through a Down‐Regulation of the Endothelial AMPK‐PI3K‐Akt‐eNOS Pathway,” Molecular Nutrition & Food Research 59 (2015): 520–532, 10.1002/mnfr.201400539. 25421217

[42] W. C. Chen , S. W. Wang , C. W. Li , et al., “Comparison of Various Solvent Extracts and Major Bioactive Components From *Portulaca Oleracea* for Antioxidant, Anti‐Tyrosinase, and Anti‐α‐Glucosidase Activities,” Antioxidants 11 (2022): 398. 35204280 10.3390/antiox11020398 PMC8869629

[43] D. A. Al‐Quwaie , A. Allohibi , M. Aljadani , et al., “Characterization of *Portulaca Oleracea* Whole Plant: Evaluating Antioxidant, Anticancer, Antibacterial, and Antiviral Activities and Application as Quality Enhancer in Yogurt,” Molecules 28 (2023): 5859. 37570829 10.3390/molecules28155859 PMC10421184

[44] I. A. M. Ahmed , F. AlJuhaimi , M. M. Özcan , N. Uslu , and E. Karrar , “Determination of the Distribution of Bioactive Compounds, Antioxidant Activities, Polyphenols and Macro and Microelement Contents in Different Parts of Wild and Cultivated Purslane (*Portulaca oleracea* L.) Plants,” Journal of Food Measurement and Characterization 19 (2025): 3714–3724, 10.1007/s11694-025-03221-w.

[45] V. Sicari , M. R. Loizzo , R. Tundis , A. Mincione , and T. M. Pellicanò , “ *Portulaca Oleracea* L. (Purslane) Extracts Display Antioxidant and Hypoglycaemic Effects,” Journal of Applied Botany and Food Quality 91 (2018): 39–46.

[46] W. Wang and Y. Kong , “Study on Chemical Constituents of *Portulaca Oleracea* L,” Journal of Liaoning Normal University:Natural Science Edition 39 (2016): 517–521.

[47] S. Wang , P. Shi , L. Qu , et al., “Bioactive Constituents Obtained from the Seeds of Lepidium Apetalum Willd,” Molecules 22 (2017): 540. 28350346 10.3390/molecules22040540 PMC6154599

[48] H. Chen , Y. Zhu , X. Zhao , and Z. Yang , “Tingli Dazao Decoction Pretreatment Ameliorates Mitochondrial Damage Induced by Oxidative Stress in Cardiomyocytes,” Journal of Ethnopharmacology 303 (2023): 115987, 10.1016/j.jep.2022.115987. 36455763

[49] Y. Liu , Z. Sun , R. Dong , et al., “Rutin Ameliorated Lipid Metabolism Dysfunction of Diabetic NAFLD via AMPK/SREBP1 Pathway,” Phytomedicine 126 (2024): 155437, 10.1016/j.phymed.2024.155437. 38394735

[50] W. Lee , S. K. Ku , and J. S. Bae , “Barrier Protective Effects of Rutin in LPS‐Induced Inflammation In Vitro and In Vivo,” Food and Chemical Toxicology 50 (2012): 3048–3055, 10.1016/j.fct.2012.06.013. 22721984

[51] M. M. J. P. E. Sthijns , P. M. Schiffers , G. M. Janssen , et al., “Rutin Protects against H_2_O_2_ ‐triggered Impaired Relaxation of Placental Arterioles and Induces Nrf2‐Mediated Adaptation in Human Umbilical Vein Endothelial Cells Exposed to Oxidative Stress,” Biochimica et Biophysica Acta (BBA)—General Subjects 1861 (2017): 1177–1189, 10.1016/j.bbagen.2017.03.004. 28286015

[52] W.‐H. Hu , D. K. Dai , B. Z.‐Y. Zheng , et al., “The Binding of Kaempferol‐3‐O‐rutinoside to Vascular Endothelial Growth Factor Potentiates Anti‐Inflammatory Efficiencies in Lipopolysaccharide‐Treated Mouse Macrophage RAW264.7 Cells,” Phytomedicine 80 (2021): 153400, 10.1016/j.phymed.2020.153400. 33157413

[53] D. Hwang , M. J. Kang , C. W. Kang , and G. D. Kim , “Kaempferol‑3‑O‑β‑Rutinoside Suppresses the Inflammatory Responses in Lipopolysaccharide‑Stimulated RAW264.7 Cells via the NF‑κB and MAPK Pathways,” International Journal of Molecular Medicine 44 (2019): 2321–2328. 31661129 10.3892/ijmm.2019.4381

[54] Y. Wang , C. Tang , and H. Zhang , “Hepatoprotective Effects of Kaempferol 3‐O‐rutinoside and Kaempferol 3‐O‐glucoside from Carthamus Tinctorius L. on CCl4‐Induced Oxidative Liver Injury in Mice,” Journal of Food and Drug Analysis 23 (2015): 310–317, 10.1016/j.jfda.2014.10.002. 28911387 PMC9351762

[55] C. Han , L. Guan , and L. Xu , “Protective Effect of Luteoloside Against Toxoplasma Gondii‐Induced Liver Injury Through Inhibiting TLR4/NF‐κB and P2X7R/NLRP3 and Enhancing Nrf2/HO‐1 Signaling Pathway,” Parasitology Research 122 (2023): 1333–1342, 10.1007/s00436-023-07833-3. 37046028

[56] K. Ji‐Eun , P. Paras Man , S. Jang , and H. K. Yi , “Anti‐Inflammatory Effect of Luteoloside Against Methylglyoxal Induced Human Dental Pulp Cells,” Journal of Applied Biomedicine 22 (2024): 33–39, 10.32725/jab.2024.002. 38505968

